# Links between core promoter and basic gene features influence gene expression

**DOI:** 10.1186/1471-2164-9-92

**Published:** 2008-02-25

**Authors:** Sandra Moshonov, Rofa Elfakess, Michal Golan-Mashiach, Hadar Sinvani, Rivka Dikstein

**Affiliations:** 1 Department of Biological Chemistry, The Weizmann Institute of Science, Rehovot 76100, Israel

## Abstract

**Background:**

Diversity in rates of gene expression is essential for basic cell functions and is controlled by a variety of intricate mechanisms. Revealing general mechanisms that control gene expression is important for understanding normal and pathological cell functions and for improving the design of expression systems. Here we analyzed the relationship between general features of genes and their contribution to expression levels.

**Results:**

Genes were divided into four groups according to their core promoter type and their characteristics analyzed statistically. Surprisingly we found that small variations in the TATA box are linked to large differences in gene length. Genes containing canonical TATA are generally short whereas long genes are associated with either non-canonical TATA or TATA-less promoters. These differences in gene length are primarily determined by the size and number of introns. Generally, gene expression was found to be tightly correlated with the strength of the TATA-box. However significant reduction in gene expression levels were linked with long TATA-containing genes (canonical and non-canonical) whereas intron length hardly affected the expression of TATA-less genes. Interestingly, features associated with high translation are prevalent in TATA-containing genes suggesting that their protein production is also more efficient.

**Conclusion:**

Our results suggest that interplay between core promoter type and gene size can generate significant diversity in gene expression.

## Background

The wide range of gene expression levels in the cell is strictly controlled by a variety of complicated mechanisms. Major contributors to gene expression rates are DNA regulatory elements that vary between individual genes. Among these sequences key roles are played by specific combinations of enhancer elements and their binding factors. Revealing additional features that influence gene expression level is central to understanding how cells work and what has been changed in diseased states.

A common element important to the overall expression level of the gene is the core promoter. The core promoter is located around the transcription start point and serves as the site of pre-initiation complex formation by RNA polymerase II and general transcription factors [1]. The TATA-box is a highly conserved core promoter element occurring in approximately 20–30% of protein encoding genes in various species [2-6]. The presence or absence of a TATA-box in core promoters has been linked in yeast [2,7-10] and human [11] to two pathways of pre-initiation complex assembly, one which is TFIID dependent (weak TATA or TATA-less) and the other is TFIID independent and SAGA dependent. A number of studies demonstrated that TATA-box occurrence or deficiency in genes is also correlated with additional traits. For example genes with a TATA box are overrepresented among inducible, stress response, developmental regulation and tissue specific genes [2,12] and TATA containing genes also show increased divergence among species, most likely due to higher rate of evolution [13]. Most recently the presence or absence of a TATA box was linked to differential regulation by transcription elongation factors [11] and the TATA box was found to be associated with higher sensitivity of gene expression to mutations [14]. Thus the core promoter is associated with features that are far beyond transcription initiation per se. In the present study we investigated possible connections between the core promoter, structural features of genes and levels of gene expression.

## Results

### Analysis of genes with canonical, weak and TATA-less promoters

To examine whether there is a link between promoters and general features of genes, we first used the DBTSS to retrieve promoters with verified TSSs and analyzed them for their core promoter type according to the presence/absence of a minimal canonical TATA box (TATAWA) at the expected location (-40 to -15 relative to the TSS) (Table 1). This definition of the TATA box is based on our recent experiments in mammalian cells and studies by others in yeast which have indicated that this is the minimal sequence that can function as a TATA box [7,10,11]. Of the 14,628 human genes that are in the DBTSS only 527 (3.6%) contain the minimal canonical TATA box; 694 genes (4.7%) have a TATA box with one mismatch (referred to as TATA-1); 3916 (27%) have a TATA with two mismatches (referred to as TATA-2) and the rest 9491 genes (65%) are TATA-less. To perform statistical analyses of the different groups we used all the genes from each group. To confirm that differences that are observed between the groups do not arise from the large difference in the number of genes between the TATA-2 and TATA-less to TATA and TATA-1, we similarly analyzed 10 randomly selected gene sets, 500 genes each, from the TATA-2 and TATA-less groups (data not shown). The results of the entire population matched that of the small groups.

**Table 1:** Analysis of DBTSS genes for core promoter type.

**Gene type**	**No. of genes**	**%**
TATA	527	3.6
TATA-1	694	4.7
TATA-2	3916	26.8
TATA-less	9491	64.9

**Total**	**14628**	**100**

### TATA-containing genes are enriched with specific biological functions

We performed functional classification of genes in the four groups (Table 2) and found that TATA and TATA-1 fall into similar functional categories that include development, response to wounding, response to external stimulus and inflammatory response. Interestingly the nucleosome and chromatin assembly category is specifically and exclusively enriched in the canonical TATA group. Considering their large number of genes, no significant enrichment of functional categories was found in the TATA-2 and TATA-less groups. These findings therefore distinguish mammalian TATA-containing groups with respect to their function as found in yeast [2].

**Table 2:** Enrichment of functional categories of genes according to core promoter type.

**Functional category**	**TATA**	**TATA-1**
Nucleosome and chromatin assembly	+	-
Development	+	+
Response to wounding	+	+
Response to external stimulus	+	+
Inflammatory response	+	+
Cell proliferation	+	-
Macromolecule biosynthesis	-	-
Protein biosynthesis	-	-
Cell cycle	-	-
Oxidative phosphorylation	-	-
RNA metabolism	-	-
Macromolecule catabolism	-	-
Cellular respiration	-	-
Cell death	-	-

### Gene length is inversely correlated with the strength of the TATA box

**Figure 1:**
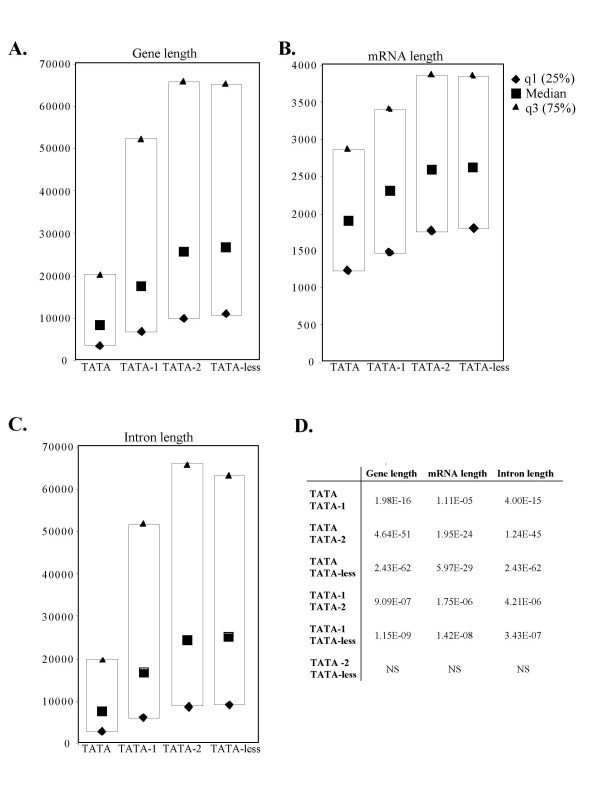
**Relationship between core promoter type and gene length.A-C. **Gene sets which differ in their core promoter, as described in the text, were analyzed for the length of their genes (A), mRNA (B) and introns (C). The boxplots present the median, 25% and 75% quartile values that were calculated from 527 TATA, 694 TATA-1, 3916 TATA-2 and 9491 TATA-less genes. **D. **The p-values of the differences in the median value between each two gene sets as indicated. NS is non-significant difference (p > 0.05).

We used the UCSC Genome Browser to retrieve structural data of genes in the different groups. We determined the median, 25% and 75% quartile gene lengths for each group and the p-values of the differences between the median values and the results are presented in boxplots (Fig. 1). Remarkably, we found that in the TATA containing groups (TATA, TATA-1 and TATA-2) gene size is inversely correlated with the compatibility to the TATA consensus, where even one nucleotide variation in the TATA box sequence results in a dramatic increase (2.1 fold p = 2 × 10^-16^) of median gene length (Fig. 1A and 1D). Likewise genes with two substitutions in their TATA or TATA-less genes are 3 fold longer than canonical TATA genes (p = 4.6 × 10^-51 ^and 2.43 × 10^-62 ^respectively) (Fig. 1A and 1D). Thus gene length is tightly associated with core promoter type.

### Gene length differences are determined by introns

The difference in size of genes with different core promoters could be due to their mature mRNA (exons), their introns or both. To examine this we compared lengths of mRNAs and introns in the TATA, TATA-1, TATA-2 and TATA-less groups. The results show small but highly significant differences in the median mRNA length between the TATA containing groups (Fig. 1B and 1D). These differences reflect the extension of the 5' and 3' untranslated regions of the non-canonical TATA and TATA-less groups (see below) rather than the coding sequences. However large differences are seen in their total intron length, up to 3.2 fold (TATA-2 vs. TATA, p = 1.2 × 10^-45^) (Fig. 1C and 1D) which are negatively correlated with the strength of the TATA. The total intron size of the TATA-less group is also longer than that of the TATA and is similar to that of the TATA-2. Thus the length of introns is the most significant variant that contributes to the difference in the gene size between the groups.

**Figure 2:**
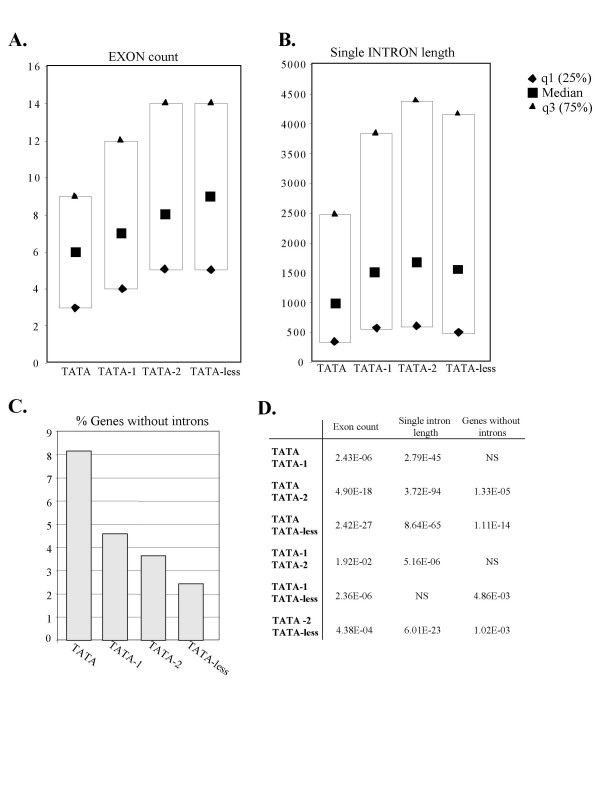
**Statistical analysis of several properties of the TATA, TATA-1, TATA-2 and TATA-less gene sets.A. **The median, 25% and 75% quartile number of exons. **B. **The median, 25% and 75% quartile length of a single intron. **C. **The fraction of intron-less genes in the different gene sets. The number of genes in each set is as in Fig. 1. **D. **The p-values of the differences in the median values (A-C) and intron-less probability (D) were calculated using the Kruskal-Wallis test with Bonferroni correction and chi-square distribution respectively. NS is a non-significant difference (p > 0.05).

We next determined the number of exons in the different groups and here again we found the exon number is inversely correlated with the strength of the TATA box (Fig. 2A and 2D). In all gene groups the length of an exon is very close (data not shown) whereas the length of an intron in the canonical TATA containing genes is significantly shorter than in non-canonical TATA or TATA-less sets (Fig. 2B and 2D). Another differential feature that we found relates to the percentage of intron-less genes which is relatively high in the TATA set and gradually declines in the TATA-1 and TATA-2 and the TATA-less groups (Fig. 2C and 2D).

### The influence of core promoter and intron size on gene expression

While the core promoter is well known for its critical role in transcription, much less is known about the impact of intron size on gene expression. A previous analysis revealed that short introns are correlated with high expression levels [15]. However, given the association of small intron size with TATA promoters, the high level of gene expression could result from the presence of a TATA promoter, the short size of introns or both. To assess the relative contribution of core promoter and intron length on gene expression we retrieved expression data for the different gene groups from the GNF Gene Expression Atlas, which has expression data from 78 human cell types. Since we wished to estimate the impact of intron size on gene expression the intron-less genes were not included in this analysis.

**Figure 3:**
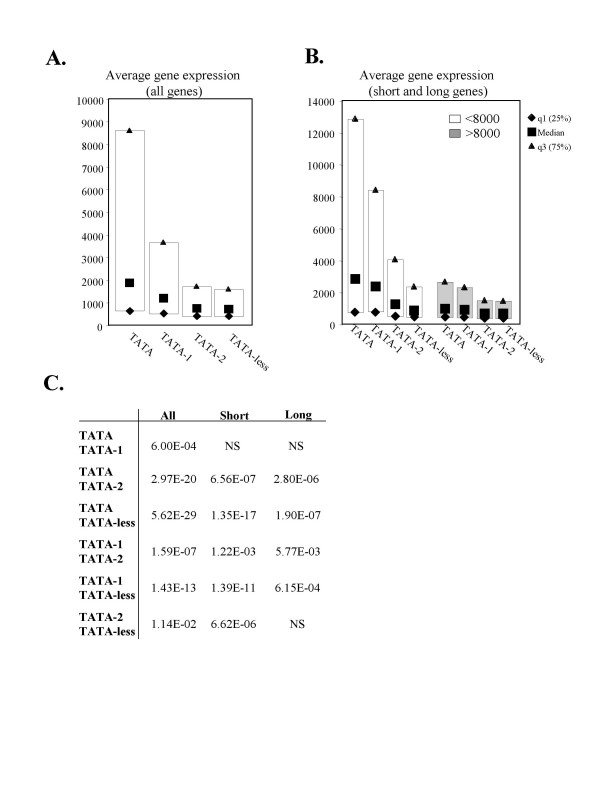
**Analysis of the expression level in the indicated gene sets.A. **The median, 25% and 75% quartiles of tissue average expression for each gene sets. **B. **The gene sets were divided into short (intron <8000 nt, white box) or long genes (intron >8000 nt, grey box), and the median, 25% and 75% quartile of the avarage expression for each gene set is shown. **C. **The p-values of the differences in the median value between each two gene sets as indicated. NS is non-significant difference (p > 0.05).

We first determined the average expression of each gene in all tissues, setting a threshold value of 200, a value that is above background. Then we determined the 25%, median and 75% quartile of the average expression for each group (Fig. 3A). Since a significant fraction of genes in the different sets shows tissue specific patterns of expression (expressed in some but not all tissues) we were concerned that the average expression from all tissues would result in a bias towards ubiquitously expressed genes rather than providing information regarding each gene's potential strength of expression. Therefore we also determined the median of the highest expression value of each gene, representing the maximal expression potential using the same threshold value of 200. The results revealed that the relative expression levels in the different groups are very similar in the two types of analysis. The data of the average expression levels is shown in Fig. 3 and that of the maximal expression is shown in Additional file 1.

The overall level of expression correlates well with the strength of the TATA box as the expression is gradually increased with the compatibility to the TATA box consensus (Fig. 3A). Thus it appears that the strength of the TATA box is highly significant for gene expression, a result consistent with previous studies showing that most variations of the TATA consensus reduce transcription of reporter genes in vitro and in vivo [16-18].

The effect of intron length on expression was determined by dividing each group into genes with short or long introns. As the TATA gene set has the shortest introns we defined genes whose intron length was less than the median intron length of the TATA (~8000) as short (Fig. 3B). A notable observation from this analysis is that long introns are associated with lower expression in all gene groups. Interestingly, this reduction in expression is less pronounced as genes deviate from the canonical TATA. For example, the difference in the median expression levels between short and long genes in the TATA-containing group is 3.3 fold (Table 3), whereas the expression of genes with TATA-less promoters is only weakly affected by intron length (1.3 fold difference, Table 3). This is confirmed by Spearman's rank correlation coefficient analysis, which shows a statistically significant negative correlation between expression levels and intron size in the four groups, with correlation of the TATA-less group being 2 fold lower than that of the TATA group (Table 4).

**Table 3:** Median expression values of short (intron length <8000) or long (intron length >8000) genes grouped according to core promoter type. *n *is the number of genes in each set, Fold is the magnitude of the difference between short and long, and *p *is the p-value of the difference.

	**Short**	**n**	**Long**	**n**	**Fold**	**p**
**TATA**	3640	199	1120	186	3.2	2.8E-05
**TATA-1**	2311	150	933	326	2.5	1.1E-05
**TATA-2**	1270	608	717	1973	1.8	5.1E-20
**TATA-less**	873	1222	690	4441	1.3	3.6E-12

**Table 4:** Correlation between gene expression (average and maximum) and intron length for each gene set. Number of genes: TATA 385, TATA-1 476, TATA-2 2581, TATA-less 5663.

	**Average expression**		**Maximal expression**	
	**Spearman Correlation**	**p-value**	**Spearman Correlation**	**p-value**

**TATA**	-0.281	2.03E-08	-0.2693	8.02E-08
**TATA-1**	-0.2554	1.65E-08	-0.2788	6.24E-10
**TATA-2**	-0.2445	2.06E-36	-0.2427	6.86E-36
**TATA-less**	-0.1447	6.92E-28	-0.1395	5.07E-26

In addition we see that the advantage of having a TATA core promoter for expression is more significant for genes bearing short introns compared to genes with long introns. For instance, the median expression of short intron genes in the TATA group is 3–4 fold higher than short intron genes from TATA-2 or TATA-less groups, but this advantage is reduced to only 1.6 fold in long genes. It therefore appears that for the TATA groups (TATA, TATA-1, TATA-2) both the strength of the TATA box and the intron length strongly influence expression. By contrast, genes with TATA-less promoters are weakly expressed regardless of their intron length. It is likely that these genes have specific features that overcome the negative effect of long introns.

### Translational features of gene groups

**Figure 4:**
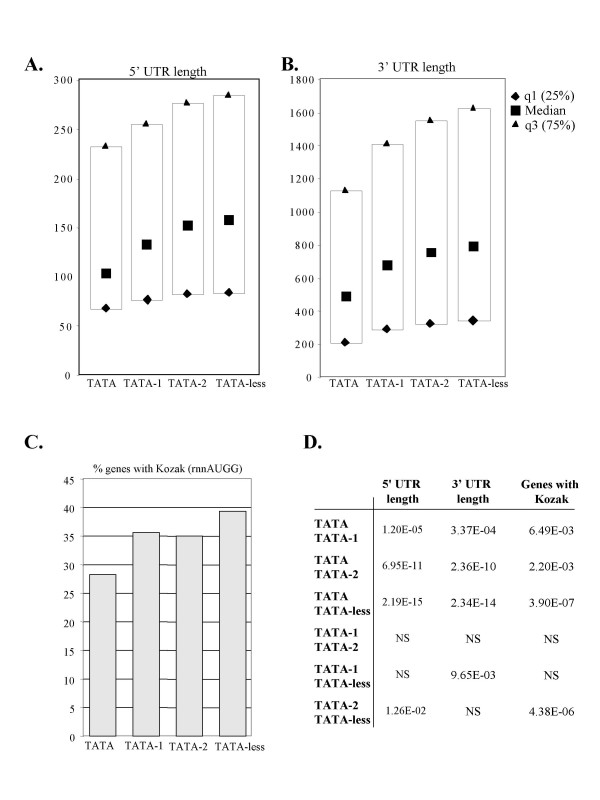
**Correlation of core promoter type with protein translation features.A. **The median, 25% and 75% quartiles lengths of the 5' UTR in each gene set. **B. **The median, 25% and 75% quartiles lengths of the 3' UTR in each gene set. **C. **The percentage of genes with the Kozak translation initiation context in each of the indicated gene set. **D. **The p-values of the differences in the median values (A&B) and Kozak probability (C) were calculated using the Kruskal-Wallis test with Bonferroni correction and chi-square distribution respectively. NS is a non-significant difference (p > 0.05).

Given that general features such as core promoter and gene size act together to affect the levels of mRNA production, we next wished to test whether there is a link between core promoter type and protein translation efficiency. Although mRNA and protein levels are generally correlated [19-23], there are also features in the mRNAs themselves that contribute to translation efficiency. Among these are the nucleotide sequence flanking the translation start codon and specific features of the 5' and 3' un-translated regions (UTR). The best characterized element that mediates high rates of translation initiation in eukaryotes is the Kozak consensus (RCCaugG, where R is A or G) [24]. Within the Kozak motif, the most important nucleotides for efficient translation initiation are the R in position -3 and the G in postion +4 relative to the initiation codon [25,26]. In addition, the length of the 5' and 3' UTR [27] were shown to affect the efficiency of translation in a negative manner. We determined the length of the 5' and 3' UTRs and the prevalence of the extended Kozak consensus RNNaugG around the translation initiation codon and in the different groups. The results show that the lengths of 5' and 3' UTRs are inversely correlated with the strength of the TATA box implying that TATA-containing genes may be translated more efficiently (Fig. 4A&B). On the other hand the frequency of a Kozak translation initiation context is higher in TATA-less genes than in the other groups (Fig. 4C).

## Conclusion

The statistical analysis of structural and functional features of mammalian genes associated with core promoter variants revealed surprisingly close ties between different steps of gene expression from transcription initiation and elongation to protein production. These findings extend further the links found in yeast between core promoter type and features that are seemingly not directly associated with transcription initiation such as gene function [2], evolution rates [13] and sensitivity of gene expression to mutations [14].

The most remarkable observation of the statistical analysis of genes divided according to their core promoters is that small variations relative to the TATA-box consensus are associated with large differences in gene length as a consequence of intron size and number. Specifically genes containing canonical TATA were found to be significantly shorter than genes bearing non-canonical TATA or TATA-less promoters. Analysis of expression of genes in the different groups highlighted several points. First, the level of expression is highly correlated with the strength of the TATA box confirming previous gene-specific studies showing that deviations from the TATA consensus reduce transcription [16-18]. Second, the expression level of the genes is affected by the type of the core promoter in a length dependent manner: long genes generally display lower levels of expression compared to short genes. However the negative effect of gene length is correlated with the strength of the TATA element, such that genes with canonical TATA are strongly suppressed, genes bearing non-canonical TATA are moderately inhibited and TATA-less genes are hardly affected by length. Third, it appears that having a TATA box in the core promoter is mostly beneficial for expression of short genes, the advantage of a strong TATA-box being diminished in long genes. We therefore propose that substantial variations in gene expression levels can be achieved through different combinations of TATA promoters with varying intron lengths. A TATA-less promoter, on the contrary, ensures similar levels of expression regardless of gene length.

Given the high cost of transcription, extending intron size in higher eukaryotes must be beneficial. Varying intron size and core promoter type may be an economic way for utilizing the same cellular constituents to modulate gene expression levels. In addition large introns with small exons may serve to reduce mutational rate in coding sequences (dilution effect). This possibility is consistent with the observations from yeast that TATA containing genes evolve at a higher rate [13,14].

The mechanistic basis underlying the links between core promoter and gene length remains to be investigated but is likely to involve RNA polymerase II, an entity present both in the core promoter and throughout the gene. Our previous findings that core promoter variants dictate recruitment of different elongation factors upon activation by NF-κB, through formation of distinct pre-initiation complexes [11], provide an initial molecular basis for the findings reported here.

## Methods

### Selection of genes and criteria for inclusion in specific core promoter group

The 14,728 *H. sapiens *genes appearing in the DBTSS version 6, with experimentally-determined transcription start site (TSS), were used in the current study. The genes were divided into groups according to their core promoter type. Core promoters with a minimal canonical TATA box (TATA), a TATA-box with one mismatch (TATA-1), a TATA-box with two mismatches (TATA-2) and the remainder of the genes (TATA-less) comprised the four gene groups. Our criteria demanded that the TATA motif (TATAWA) and its alternatives were strictly located between -40 and -15 with respect to the TSS. Using the 'pattern matching' tool of the Regulatory Sequence Analysis Tools (available on the internet) we were able to direct each gene to its appropriate group. The TATA, TATA-1, TATA-2 and TATA-less groups finally comprised 527, 694, 3916 and 9491 genes respectively.

### Measurements of basic gene features

Genes were classified according to their function using the gene-annotation enrichment analysis (DAVID Bioinformatics Resources 2007). The data of the various gene features were retrieved from the UCSC genome browser March 2006 assembly. In this browser we used the 'Tables' to obtain coordinates of different tracks (e.g. gene start, gene end, exon count, UTRs exons etc.) in order to calculate the following features: gene length, mRNA length, intron length, exon number, 5' UTR length and 3' UTR length with the Excel program. UCSC Table Browser was also used to retrieve DNA sequences of the translation initiation site of each mRNA which was scanned for the presence of the Kozak motif (RNNAUGG).

### Gene expression

Gene expression data (gcRMA) for each group of genes was downloaded from SymAtlas v1.2.4 (available on the internet at the Novartis Research Foundation site). For each gene only the major probe set was used. Expression values below 200 were considered background and omitted from the analysis. The average and maximum expression values of each gene were calculated. Genes were then divided into two sets according to their intron size (less or more than 8000 nt) and the median, 25% and 75% quartiles of average expression of each set were determined and are presented as boxplots.

### Statistical analyses of gene features

The range of measurements for each gene feature within the groups was wide, making it statistically inappropriate to compare their means, particularly since we did not wish to exclude any gene that did not appear to fit the general observed pattern. We therefore determined the 25%, median and 75% quartile values for the different features. We measured the significance of the differences between the median values (6 groups) by the Kruskal-Wallis test with the Bonferroni correction using the Statistics Toolbox of the MATLAB program (The MathWorks). The Spearman's rank correlation coefficient analysis between expression levels and intron size was performed using the MATLAB program. The likelihood of there being significant differences between the groups regarding the absence of introns and the presence of the Kozak motif sequences was calculated using the Chi-square test.

## Authors' contributions

SM participated in the design of this study, performed the bioinformatic and statistical analyses of the various gene features and wrote this paper. RE participated in the bioninformatic analysis of core promoters. MG performed the bioinformatic analysis of the gene expression data. HS participated in the intellectual and statistical analyses. RD conceived the study, participated in its design, analyzed the data and wrote the paper. All authors read and approved the final manuscript.

## Supplementary Material

Additional file 1 Analysis of the maximal expression level in gene sets. **A. **The gene sets were divided into all (white bars) short (intron < 8000 nt, black bars) or long genes (intron > 8000 nt, striped bars), and the median of the maximal expression for each gene set is shown. The numbers show the fold change between short and long in each group. **B. **The p-values of the differences in the median value between each two gene sets as indicated. NS is non-significant difference (p > 0.05). Click here for file
