# Supplementary material for: Links between core promoter and basic gene features influence gene expression
Source: BMC Genomics. 2008 Feb 25;9:92. doi: 10.1186/1471-2164-9-92 (PMC2279122; doi:10.1186/1471-2164-9-92)
Supplement: Additional file 1 — Analysis of the maximal expression level in gene sets. A. The gene sets were divided into all (white bars) short (intron < 8000 nt, black bars) or long genes (intron > 8000 nt, striped bars), and the median of the maximal expression for each gene set is shown. The numbers show the fold change between short and long in each group. B. The p-values of the differences in the median value between each two gene sets as indicated. NS is non-significant difference (p > 0.05). [file 1471-2164-9-92-S1.ppt]

## Slide 1
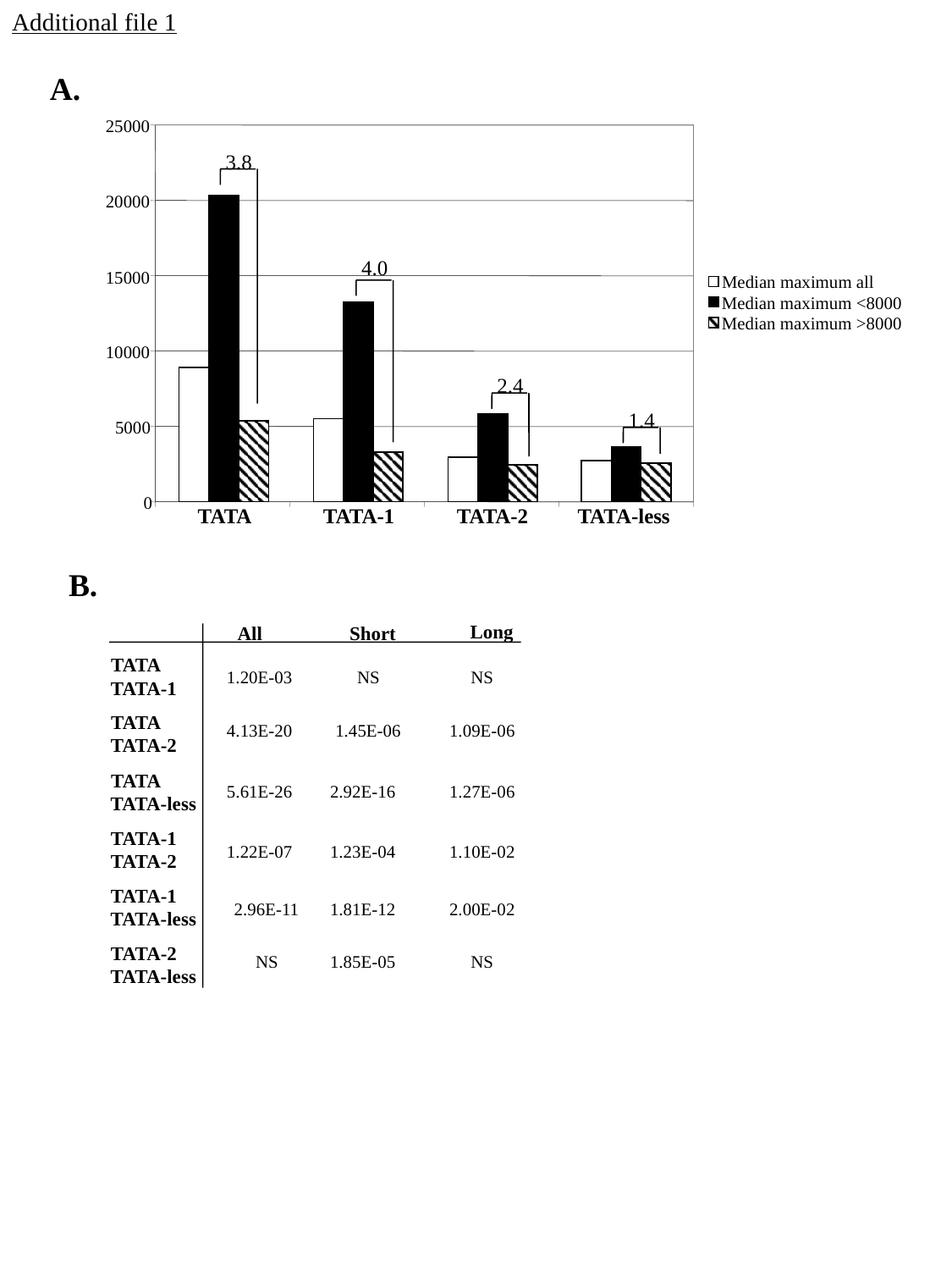

Additional file 1
A.
25000
3.8
20000
4.0
15000
Median maximum all
Median maximum <8000
Median maximum >8000
10000
2.4
1.4
5000
0
TATA
TATA-1
TATA-2
TATA-less
B.
Long
All
Short
TATA
TATA-1
TATA
TATA-2
TATA
TATA-less
TATA-1
TATA-2
TATA-1
TATA-less
TATA-2
TATA-less
1.20E-03
NS
NS
4.13E-20
1.45E-06
1.09E-06
5.61E-26
2.92E-16
1.27E-06
1.22E-07
1.23E-04
1.10E-02
2.96E-11
1.81E-12
2.00E-02
NS
1.85E-05
NS
